# Ambrisentan ameliorates methotrexate nephrotoxicity through modulation of RhoA/ROCK and PI3K/MDM2-associated signaling: network pharmacology-guided discovery and in vivo confirmation

**DOI:** 10.3389/fphar.2026.1976309

**Published:** 2026-10-29

**Authors:** Sara A. Baraka, Nermin Eissa, Marwa M. Nagib, Asmaa M. Elmaghraby, Azza A. K. El-Sheikh, Samar F. Darwish

**Affiliations:** 1 Department of Pharmacology and Toxicology, Faculty of Pharmacy, Ahram Canadian University, Cairo, Egypt; 2 Department of Biomedical Sciences, College of Health Sciences, Abu Dhabi University, Abu Dhabi, United Arab Emirates; 3 Department of Pharmacology and Toxicology, Faculty of Pharmacy, Misr International University, Cairo, Egypt; 4 Department of Histology and Cell Biology, Faculty of Medicine for Girls, Al-Azhar University, Cairo, Egypt; 5 Basic Health Sciences Department, College of Medicine, Princess Nourah bint Abdulrahman University, Riyadh, Saudi Arabia; 6 Pharmacology & Toxicology Department, Faculty of Pharmacy, Badr University in Cairo (BUC), Cairo, Egypt

**Keywords:** acute kidney injury, ambrisentan, MDM2, methotrexate, network analysis, PPAR-γ

## Abstract

**Introduction:**

Methotrexate (MTX) is a potent antineoplastic agent; however, its clinical application is frequently limited by nephrotoxicity, which may result in treatment interruption. Previous evidence indicates that MTX can promote endothelin-1 (ET-1)-associated renal injury. Therefore, this study investigated whether blocking ET-1 receptors with Ambrisentan (AMB) could alleviate MTX-induced acute kidney injury (AKI).

**Methods:**

Network pharmacology analysis was performed to identify potential targets and pathways associated with AMB and MTX-induced AKI. For *In-vivo* validation, twenty-four rats were divided into four groups: control, AMB, MTX, and MTX+AMB.

**Results and Discussion:**

Network pharmacology identified 102 overlapping targets between AMB and MTX-induced AKI, with 20 hub genes identified through protein–protein interaction network analysis, highlighting PPAR-γ as a key target. KEGG pathway enrichment revealed the involvement of RhoA/ROCK and PI3K-associated signaling pathways, in correlation with inflammatory and apoptotic markers. Experimentally, AMB co-treatment improved renal function parameters and histopathological alterations, restored oxidative balance, and attenuated MTX-induced inflammatory and apoptotic responses, including ICAM-1, NF-κB, RhoA/ROCK, Bax, Caspase-3 and p53. Furthermore, AMB restored the expression of PPAR-γ, PI3K, MDM2, and Bcl-2, suggesting the involvement of PI3K/MDM2-associated signaling alterations in AMB-mediated nephroprotection. In conclusion, AMB may mitigate MTX-induced nephrotoxicity through modulation of oxidative stress, inflammation, and apoptosis. Further studies are required to evaluate its effects on MTX pharmacokinetics, renal elimination, antineoplastic efficacy, and therapeutic potential in established MTX-induced AKI.

## Graphical Abstract

**Figure:**
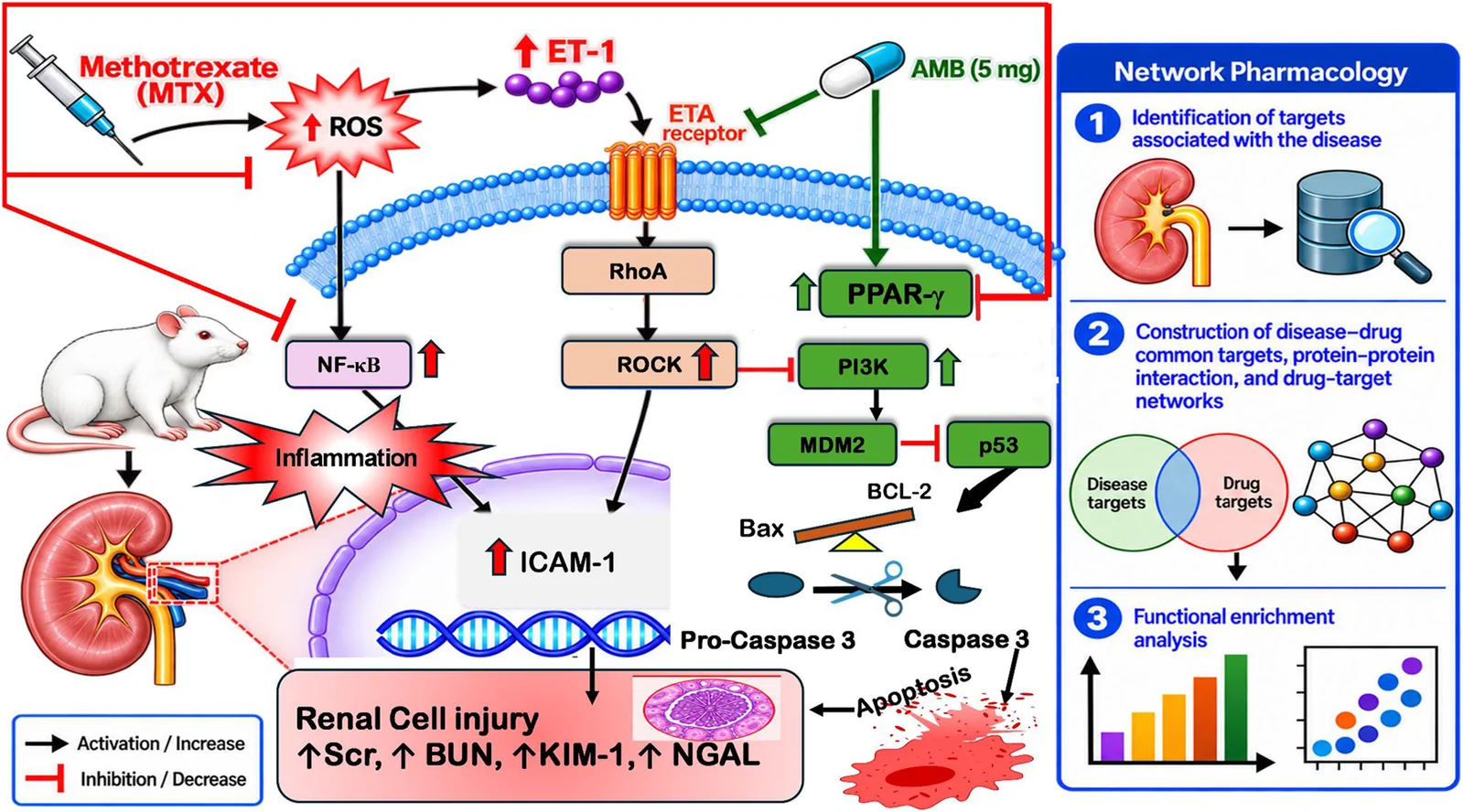

## 1 Introduction

Methotrexate (MTX), a cytotoxic antifolate agent, is widely used in the clinical treatment of different types of cancers and various inflammatory diseases (Pannu, 2019; Türk et al., 2022). However, the risk of nephrotoxicity linked to its use remains a major challenge that restricts its clinical applicability (Donaldson Dasgupta et al., 2023). MTX-induced renal damage spans a spectrum from mild subclinical tubulopathy to overt acute kidney injury (AKI) (Arab et al., 2022). Compelling data reveal that MTX stimulates the expression of endothelin-1 (ET-1), provoking pronounced pro-inflammatory, pro-oxidative, and pro-fibrotic responses (Kowalczyk et al., 2015; Al-Wakeel et al., 2026). ET-1 is a potent vasoactive peptide that is produced primarily by endothelial cells, and also by renal epithelial and mesangial cells (Barton and Yanagisawa, 2008), in which blocking its effect has protected the kidney against Ischemia reperfusion-induced AKI (Guan et al., 2015). These observations support the pursuit of ET-1/ETAR-modulating therapies in mitigating renal injury induced by MTX.

In this context, Ambrisentan (AMB), a diphenyl propionic acid derivative, serves as a highly selective ETAR antagonist and is currently approved for therapeutic use in pulmonary arterial hypertension (Zancan et al., 2021). Preclinical evidence indicates that AMB exerts renoprotective effects by reducing kidney apoptosis and inflammation in a rat model of I/R injury (Kazimoglu et al., 2020). However, its specific role in MTX-induced nephrotoxicity has not yet been fully elucidated.

Peroxisome proliferator-activated receptor-gamma (PPAR-γ) is a ligand-activated transcription factor belonging to the nuclear receptor superfamily, that regulates multiple physiological processes. PPAR-γ was reported to suppress oxidative stress and inflammation, thus controlling renal injury (Huang et al., 2021). Additionally, emerging evidence indicates a functional interplay between endothelin signaling and PPAR-γ regulation. Furthermore, RhoA is the most prominent protein that belongs to the Ras superfamily, and its activation stimulates Rho-kinase (ROCK), which mediates downstream cellular responses (Boettner and Van Aelst, 2002). Notably, pharmacological inhibition of ROCK isoforms has been shown to suppress inflammation and improve renal function in experimental models of AKI (Şahin et al., 2025). Additionally, ET-1 evidenced to activate the RhoA/ROCK signaling cascade through ETA receptor engagement, establishing this pathway as a key downstream effector of ET-1 activity in renal pathology (Saleh et al., 2023).

In light of the foregoing data, the current study sought to examine the protective role of AMB against MTX-induced nephrotoxicity and to explore its mechanistic basis based on the hypothesis that pharmacological blockade of ET-1/ETAR signaling may counteract ET-1–mediated suppression of PPAR-γ activity, thereby enhancing its anti-inflammatory and antioxidant functions. Given the potential for AMB repurposing, its use as a prophylactic adjunct during MTX therapy warrants further investigation. Its potential utility as an early intervention after the onset of renal injury also remains to be established. The present study therefore evaluated AMB administered before and during MTX exposure as a first step toward establishing its renoprotective potential.

## 2 Materials and methods

### 2.1 Drugs and chemicals

Methotrexate (20 mg/kg) was supplied by Johnlee Pharmaceuticals (Mumbai, India) and administered as a single intraperitoneal injection. Ambrisentan, obtained from AUG Pharma (sixth Industrial Zone, sixth of October City, Egypt), was prepared in 0.5% carboxymethyl cellulose (CMC) provided by Al Gomhorya, Cairo, Egypt, and was administered orally for 10 consecutive days at a dose of 5 mg/kg/day. All other chemicals and reagents utilized in the study were of high analytical grade quality.

### 2.2 Analysis of network pharmacology

#### 2.2.1 Obtaining the molecular targets associated with ambrisentan and methotrexate

The chemical structures of AMB and MTX, in Canonical SMILES formats, were obtained from PubChem database (Supplementary Table S1), (https://pubchem. ncbi.nlm.nih.gov/, accessed on 3 February 2026) (National Center for Biotechnology Information, 2024). Five datasets were subsequently examined to identify the targets for AMB as follows: (1) The SwissTargetPrediction database (https://www.swisstargetprediction.ch/result.php?job=1723967044&organism=Homo_sapiens, accessed on 3 February 2026) (Daina et al., 2019); (2) Comparative Toxicogenomics Database (CTD), https://ctdbase.org/detail.go?type=chem&acc=C467894, accessed on 3 February 2026) (Davis et al., 2023); (3) The Targetnet database (http://targetnet.scbdd.com/calcnet/calc_text/, accessed on 3 February 2026) (Yao et al., 2016); (4) The Similarity ensemble approach (SEA) database (https://sea.bkslab.org/, accessed on 3 February 2026) (Keiser et al., 2007); and (5) The STITCH database (https://stitch-db.org/, accessed on 3 February 2026) (Szklarczyk et al., 2016).

Similarly, four datasets were also examined to identify the targets for MTX as follows: (1) The SwissTargetPrediction database (https://www.swisstargetprediction.ch/result.php?job=1315485240&organism=Homo_sapiens, accessed on 3 February 2026) (Daina et al., 2019); (2) Comparative Toxicogenomics Database (CTD), https://ctdbase.org/detail.go?type=chem&acc=D008727, accessed on 3 February 2026) (Davis et al., 2023); (3) The Targetnet database (http://targetnet.scbdd.com/calcnet/calc_text/, accessed on 3 February 2026) (Yao et al., 2016); and (4) The Similarity ensemble approach (SEA) database (https://sea.bkslab.org/, accessed on 3 February 2026) (Keiser et al., 2007).

For further investigation, the targets obtained from the five databases were standardized into valid gene symbols using the *UniProt* database (https://www.uniprot.org/, accessed on 3 February 2026) (Bateman et al., 2023). Through using Jvenn tool (https://jvenn.toulouse.inrae.fr/app/example.html, accessed on 3 February 2026) (Bardou et al., 2014), all duplicate targets were removed. The empirical targets of AMB and MTX were inputted into an online Jvenn software to create Venn diagrams.

#### 2.2.2 Identification of targets associated with the disease

The following keywords, “renal tubular dysfunction” and “nephrotoxicity” were used to retrieve the molecular targets linked to the target diseases from five databases (accessed on 3 February 2026): (1) GeneCards database (https://www.genecards.org/) (Stelzer et al., 2016); (2) The Open Targets Platform database, (https://platform.opentargets.org/) (Ochoa et al., 2023); (3) CTD, (https://ctdbase.org/detail.go?type=disease&acc=MESH%3AD005198) (Davis et al., 2023); (4) MalaCards database (https://www.malacards.org/) (Rappaport et al., 2017) and (5) The Online Mendelian Inheritance in Man (OMIM) (https://www.omim.org/) (Amberger and Hamosh, 2017). The five databases provide supplementary resources for acquiring targets applicable to illnesses. All targets were converted into the uniform gene symbol format using *UniProt* and any duplicate targets were eliminated.

#### 2.2.3 Construction of disease-drug common targets, protein–protein interaction, and drug-target networks

To obtain a disease-target set specifically relevant to MTX-induced renal injury, we intersected the broad disease-target list (‘nephrotoxicity’ and ‘renal tubular dysfunction/injury’) with the MTX-associated target list using the Jvenn online tool (https://jvenn.toulouse.inrae.fr/app/example.html) (Bardou et al., 2014) with a default confidence level of >0.4. The organism selected for the analysis was *Homo sapiens*. Only genes present in both the MTX target set and the disease target set were retained as MTX–renal-injury-associated targets and used for subsequent overlap with AMB targets, PPI network construction, and enrichment analysis. A second Venn diagram was constructed between the resulting previous intersection targets of MTX-Disease and the targets of AMB, using the same steps.

To construct the protein–protein interactions (PPI) network of the intersected targets, the final related targets were uploaded to the STRING database Version 12.0 (https://string-db.org/, accessed on 5 February 2026) (Szklarczyk et al., 2023). The network data were downloaded as a “TSV” format file for subsequent analysis, and a visualized network image was downloaded in high-resolution PNG format.

Following the construction of the PPI network, a compound-target network was also established to connect AMB with the overlapping targets. The Cytoscape 3.10.4 program (NIGMS, United States) (Shannon et al., 2003) was employed to visualize the networks. Then, the common overlapped targets were filtered by degree value and subsequently, ranked based on Betweenness and Closeness by using Cytoscape plugin CytoNCA (Tang et al., 2015). The top 20-ranked proteins were identified as hub targets. This strategy is consistent with numerous recent network pharmacology studies that prioritize hub genes for functional enrichment to identify core signaling pathways (Huang et al., 2022; Qian and Wang, 2026).

#### 2.2.4 Functional enrichment analysis

The Gene Ontology (GO) analysis and Kyoto Encyclopedia of Genes and Genomes (KEGGs) pathway enrichments were conducted using the ShinyGO software (http://bioinformatics.sdstate.edu/go/#tab-6415-3, accessed on 6 February 2026) (Ge et al., 2020). A critical P value of less than 0.05 was used as the cutoff value. GO molecular function (MF), GO cellular component (CC), GO biological process (BP), and KEGG were selected as data sources. The results were illustrated in the form of lollipop plots for GO terms and bar graph for KEGG pathway supplied by the ShinyGO website.

### 2.3 Animals

Twenty-four male albino rats (aged 5–6 weeks; body weight 150–200 g) were obtained from El Nile company (Cairo, Egypt). The animals were acclimatized for a week before experimentation under standard laboratory conditions at a temperature of 25 °C ± 1 °C and relative humidity of 55% ± 5% with alternating 12-h light and dark cycles with free access to standard diet pellets and tap water *ad libitum*. The protocol was approved by the Research Ethics Committee (REC) of the Faculty of Pharmacy, Badr University in Cairo, Egypt, which strictly adheres to the Guidelines for Care and Use of Laboratory Animals (National Research Council, 2011); permit number: BUC-IACUC/PHA/202/A/2026.

### 2.4 Experimental design

Rats were randomly divided into four groups (n = 6). Our experimental procedures comply with the ARRIVE guideline. As illustrated in Figure 1, the control group received 0.5% CMC orally once daily for 10 days, whereas the AMB control group was treated with AMB (5 mg/kg, p.o.) once daily for the same duration (Almutary et al., 2025). The MTX group was given 0.5% CMC orally once daily for 10 days and administered a single IP dose of MTX (20 mg/kg) on day 7 (Mishriki et al., 2024). The MTX + AMB group received oral AMB (5 mg/kg) once daily for 10 days in combination with a single IP injection of MTX (20 mg/kg) on the seventh day. Blood samples were collected on the 11th day from the retro-orbital plexus under thiopental anesthesia (50 mg/kg, i.p) (Liu and Yao, 2005), as approved under the institutional animal-use protocol (BUC-IACUC/PHA/202/A/2026). The terminal sampling approach was selected to obtain the serum volume required for the planned renal-function and biomarker analyses at the terminal experimental time point. The procedure was performed by trained personnel in accordance with the approved protocol, with measures taken to minimize potential ocular trauma and animal discomfort, and serum was separated by centrifugation at 5000 rpm for 10 min, in order to measure renal performance indices. Subsequently, rats were euthanized by cervical dislocation; kidney specimens were fixed in 10% neutral buffered formalin for histopathological examination, while the remaining tissues were stored at −80 °C for further biochemical analysis.

**FIGURE 1:**
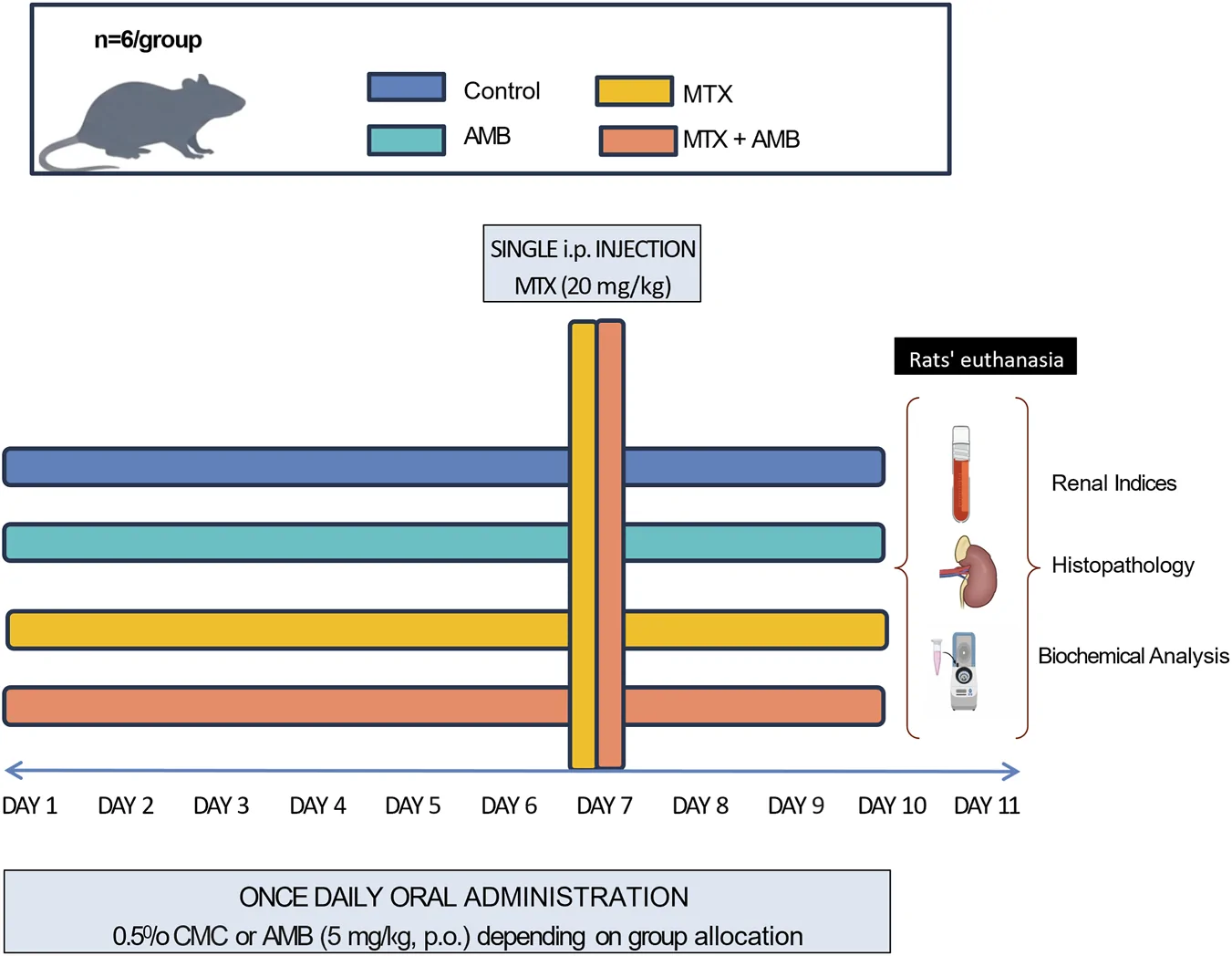
Schematic representation of the experimental design. Rats were randomly allocated into four groups (n = 6 per group). Ambrisentan (AMB; 5 mg/kg, p.o.) or vehicle (0.5% CMC, p.o.) was administered once daily for 10 consecutive days. Methotrexate (MTX; 20 mg/kg, i.p.) was given as a single injection on Day 7. On Day 11, blood and kidney samples were collected for the evaluation of renal function, histopathological alterations, and biochemical parameters. AMB: Ambrisentan, CMC: carboxymethyl cellulose, MTX: Methotrexate.

### 2.5 Histopathologic examination using H & E stain

Fixed kidney tissue in buffered formalin was embedded in paraffin and sectioned into 5 μm slices using a rotary microtome (Leica RM 2125 RM, Leica Microsystems, Nussloch, Germany). Following deparaffinization, the slides were stained with Hematoxylin and Eosin (H&E) for general histological assessment. Stained sections were meticulously examined, photographed, and subjected to morphometric analysis using a light microscope (Leica Qwin, Cambridge, UK) paired with image analysis software. For each rat, six randomly selected, non-overlapping high-power fields (×400) were examined. A single semiquantitative histological score was assigned per animal based on the following kidney damage criteria: glomerular atrophy, vacuolar degeneration of tubular epithelial cells, desquamation, loss of the brush border, formation of hyaline casts, infiltration of inflammatory cells, and vascular congestion were. Kidney histological changes were scored from (0–4) with the following semiquantitative scale: 0: no histological changes and the tissues appeared normal; 1: histological changes in less than 25% studied microscopic domains; 2: histological changes in 26%–50% studied microscopic domains; 3: histological changes in 51%–75% studied microscopic domains; and 4: histological changes in more than 75% studied microscopic domains (Gibson-Corley et al., 2013). These per-animal scores were used for statistical analysis; individual microscopic fields were not treated as independent observations.

### 2.6 Histopathological examination using PAS stain

Additionally, Periodic Acid-Schiff (PAS) staining was performed following deparaffinization to highlight mucopolysaccharides within the renal tissue and to visualize glomerular and tubular basement membranes, as well as the apical brush borders of the tubules (Suvarna et al., 2018). Stained sections were meticulously examined, photographed, and subjected to morphometric analysis using a light microscope (Leica Qwin, Cambridge, UK) paired with image analysis software. The optical density of PAS-positive reaction at the apical brush borders of proximal convoluted tubules in PAS-stained sections were determined (Nasralla et al., 2021). Furthermore, Histological findings were additionally graded from 0 to 4 (0, no change; 1, changes affecting 25% of the field of view; 2, changes affecting 25%–50%; 3, changes affecting 51%–75%, and 4, changes affecting >75% of the field), according to the area affected by the features of interest (absent/deranged brush border in proximal tubular epithelia, thickening and irregularity of the basement membrane of the renal tubules). All image acquisition and quantitative analyses were performed by two investigators who were blinded to the treatment groups.

### 2.7 Immunohistochemical assessment

The expression of Bcl-2, Bax, p53, and cleaved caspase-3 in renal tissue was evaluated by immunohistochemical staining. Mouse monoclonal primary antibodies against Bcl-2, Bax, and p53 (1:100 dilution; Santa Cruz Biotechnology, Inc., Dallas, TX, USA; cat. nos. sc-509, sc-7480, and sc-126, respectively), and a rabbit polyclonal antibody against cleaved caspase-3 (1:200 dilution; Cell Signaling Technology, Danvers, MA, USA; cat. no. #9661) were utilized. In summary, the procedure began with deparaffinization of the slides, followed by antigen retrieval. To neutralize endogenous peroxidase activity, the slides were treated with 3% H2O2 in methanol for 15 min, then rinsed with Tris-buffered saline (TBS). Next, normal goat serum was applied for 30 min at room temperature to block non-specific binding of immunoglobulins. Primary antibodies were incubated overnight at 4 °C and subsequently washed with TBS. Following this, the slides were exposed to biotinylated secondary antibodies for 1 h at room temperature. After another TBS rinse, the sections were processed using an avidin-biotin-peroxidase complex for 30 min and subsequently stained with diaminobenzidine substrate. Counterstaining was accomplished using hematoxylin. In negative control slides, all the above steps were carried out, except for the application of the primary antibodies. Thereafter slides were dehydrated, cleared, and mounted for light microscopy examination and examined blindly. The mean area percentage of brown, cytoplasmic immunopositive cells of Bax and Bcl-2 in the immunohistochemically treated sections was calculated, irrespective of the staining intensity. Glomeruli and large vessels were excluded from the image analysis (Nasralla et al., 2021). Meanwhile, the mean number of both p53 and cleaved caspase-3 immunopositive cells of the renal tissue in different experimental groups was determined (Topcu‐Tarladacalisir et al., 2016). All image acquisition and quantitative analyses were performed by two investigators who were blinded to the treatment groups.

### 2.8 Renal function assessment and kidney injury biomarker analysis

Renal function was assessed by measuring serum creatinine and urea levels using commercially available assay kits according to the manufacturers’ instructions. Serum creatinine was quantified using a rat-specific ELISA kit (Catalog No. E4370-100, BioVision, Milpitas, CA, USA) according to (Mohamed et al., 2024), while urea concentration was determined using a colorimetric assay kit (Catalog No. K376-100, BioVision, Milpitas, CA, USA) based on a chromogenic reaction. In parallel, renal injury biomarkers, including neutrophil gelatinase-associated lipocalin (NGAL) and kidney injury molecule-1 (KIM-1), were measured using rat-specific ELISA kits. NGAL levels were determined using a Rat NGAL ELISA Kit (Catalog No. SEB388Ra, Cloud-Clone Corp., Houston, TX, USA), whereas KIM-1 levels were assessed using a Rat KIM-1 ELISA Assay Kit (Catalog No. RKM29-K01, Eagle Biosciences Inc., Nashua, NH, USA). For ELISA assays, standards and serum or tissue homogenate samples were added to antibody pre-coated microplates, followed by incubation with biotinylated detection antibodies and HRP-streptavidin conjugate. After washing steps, TMB substrate solution was added for color development, and the reaction was terminated using stop solution. Absorbance was measured spectrophotometrically at 450 nm using a microplate reader. Biomarker concentrations were calculated from the corresponding standard calibration curves according to the manufacturers’ protocols.

### 2.9 Assessment of oxidative stress and redox status

To evaluate the effect of Ambrisentan on renal oxidative stress status, oxidative stress–related biomarkers were measured in kidney tissue homogenates. Malondialdehyde (MDA), a marker of lipid peroxidation, was quantified using a rat-specific ELISA kit (Catalog No. ER 1878, FineTest®, Wuhan Fine Biotech Co., Wuhan, China). Superoxide dismutase-1 (SOD1), an indicator of antioxidant defense activity, was measured using a rat-specific assay kit (Catalog No. E4584-100, BioVision, Milpitas, CA, USA). Kidney tissues were homogenized in ice-cold phosphate-buffered saline to minimize oxidative degradation and preserve enzymatic activity. The homogenates were centrifuged at 4 °C to remove cellular debris, and the resulting supernatants were collected for biochemical analyses. All assays were performed according to the manufacturers’ instructions. Absorbance was measured at 450 nm using a microplate reader, and analyte concentrations were calculated using standard calibration curves generated for each assay.

### 2.10 Assessment of ICAM-1, PPAR-γ, and NF-κB markers

The effects of Ambrisentan on inflammatory and transcriptional regulators were evaluated through both protein and gene expression analyses. Renal ICAM-1 protein levels were quantified in kidney tissue homogenates using a Rat ICAM-1/CD54 ELISA Kit (Catalog No. E-EL-R2850, Elabscience Biotechnology Inc., Houston, TX, USA) according to the manufacturer’s instructions. For gene expression analysis, total RNA was extracted from renal tissue homogenates using the Direct-zol™ RNA Miniprep Plus Kit (Catalog No. R2072, Zymo Research Corp., Irvine, CA, USA). RNA concentration and purity were assessed spectrophotometrically using absorbance ratios at 260/280 nm. Relative PPAR-γ gene expression was evaluated using the SuperScript™ IV One-Step RT-qPCR System (Catalog No. 12594100, Thermo Fisher Scientific, Waltham, MA, USA) with gene-specific primers. Reverse transcription and quantitative PCR amplification were performed sequentially in a single reaction tube. Thermal cycling conditions included reverse transcription at 55 °C for 15 min, initial denaturation at 95 °C for 2 min, followed by 40 amplification cycles consisting of denaturation at 95 °C for 10 s, annealing at 55 °C for 10 s, and extension at 72 °C for 30 s, with a final extension step at 72 °C for 5 min. Relative gene expression levels were normalized to GAPDH as the housekeeping gene and calculated using the 2^−ΔΔCT^ method. Renal NF-κB protein levels were determined using a commercial ELISA kit (Catalog No. EIA0591, Wuhan Fine Biotech Co., Ltd., Wuhan, China) according to the manufacturer’s protocol.

### 2.11 Assessment of RhoA/ROCK, PI3K, and MDM2 levels

To investigate whether Ambrisentan modulates the RhoA/ROCK signaling pathway, renal tissue levels of RhoA and ROCK1 proteins were quantified using rat-specific ELISA kits (RhoA: Catalog No. ER 2078, FineTest®, Wuhan Fine Biotech Co., Wuhan, China; ROCK1: Catalog No. A79845, antibodies.com, Cambridge, UK). PI3Kα and MDM2 protein levels were also measured using rat ELISA kits (PI3Kα: Catalog No. ER 1910, FineTest®, Wuhan Fine Biotech Co., Wuhan, China; MDM2: Catalog No. MBS096918, MyBioSource, San Diego, CA, USA). All ELISA assays were performed according to the manufacturers’ instructions using sandwich ELISA methodology. Briefly, standards and tissue homogenate samples were added to antibody pre-coated wells, followed by incubation with detection antibodies and HRP-conjugated reagents. After washing steps, TMB substrate was added for chromogenic detection, and the reaction was terminated using stop solution. Optical density was measured at 450 nm using a microplate reader, and protein concentrations were calculated from the generated standard curves.

### 2.12 Statistical analysis

The GraphPad Prism® software package version 8 (GraphPad Software Inc., CA, USA) was used for graphical data representation and statistical analyses. For parametric data, results were expressed as mean ± SD, and a one-way analysis of variance (ANOVA) was performed, followed by Tukey’s *post hoc* for multiple comparisons. For non-parametric data, results were expressed as median (interquartile range), and Kruskal–Wallis test followed by Dunn’s multiple comparison test were performed.

## 3 Results

### 3.1 The network pharmacology analysis

#### 3.1.1 Identifying the targets associated with AMB and MTX

To identify the targets linked to AMB, we analyzed five databases. 234 targets in total were found using the following databases: SwissTargetPrediction (100), SEA (16), CTD (14), STITCH (3), and Targetnet (101). Nevertheless, once duplicates were eliminated, they were downsized to 208 targets (Supplementary Table S2).

Four datasets were employed to determine the molecular targets linked to MTX. A total of 2846 targets were acquired, with SwissTargetPrediction (100), SEA (46), CTD (2680), and Targetnet (20). The number of final targets declined to 2775 following the process of integrating and eliminating the duplicates (Supplementary Table S3).

#### 3.1.2 Identifying the targets associated with MTX-induced kidney injury

The molecular targets associated with the diseases were retrieved by utilizing five databases; a total of 22,732 targets were obtained: 20,741 from CTD, 1264 from GeneCards, 564 from MalaCards, 142 from Open Targets Platform and 21 from OMIM. Finally, 21,427 targets remained after removing repetitions (Supplementary Table S4). The constructed Venn diagram showed the intersection targets between both MTX and Disease final lists, in which among the 2775 MTX targets and the 20,741 disease targets, 2542 unique targets were identified for MTX-induced kidney injury (Figure 2A; Supplementary Table S5).

**FIGURE 2:**
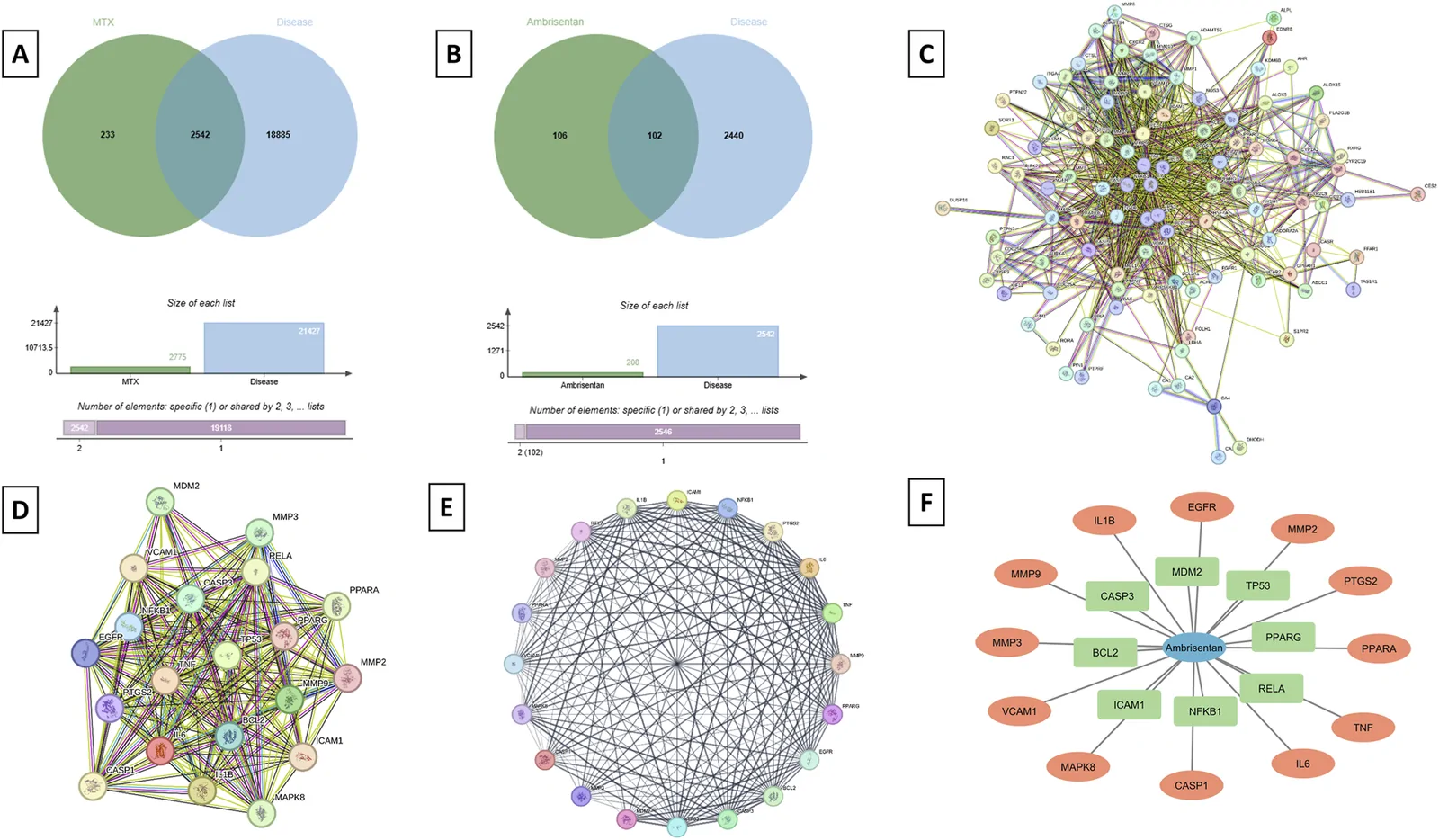
Network pharmacology analysis. **(A)** The 2542 intersection targets overlap MTX with “renal tubular dysfunction” and “nephrotoxicity” **(B)** The 102 intersection targets overlap AMB with MTX-induced renal injury, **(C)** A protein-protein interaction (PPI) network consisting of 101 nodes connected by 872 edges. **(D)** The PPI network of 20 hub targets (IL6, TNF, IL1B, TP53, MMP9, CASP3, BCL2, NFKB1, EGFR, PTGS2, MMP2, PPARG, ICAM1, VCAM1, CASP1, RELA, PPARA, MAPK8, MDM2, MMP3), **(E)** Circular layout of the drug-20 hub targets network showing the interaction line between nodes, **(F)** Concentric layout of the drug-20 hub targets network: Blue ellipse: Ambrisentan, Green rectangle: the experimentally validated hub targets (MDM2, TP53, BCL2, NFKB1, RELA, PPARG, ICAM1, CASP3), Orange ellipse: the rest of hub targets (IL6, TNF, IL1B, MMP9, EGFR, PTGS2, MMP2, VCAM1, CASP1, PPARA, MAPK8, MMP3).

#### 3.1.3 Potential targets of AMB overlapped with MTX-induced kidney injury

A second Venn diagram was constructed between the resulting previous intersection targets between (MTX and Disease final lists) and the targets of (AMB), in which among the 2542 targets for MTX-induced kidney injury and the 208 targets for AMB, 102 overlapped unique targets were determined (Figure 2B; Supplementary Table S5).

#### 3.1.4 Protein–protein interaction (PPI) network of the 102 AMB-Disease overlapped targets

A PPI diagram was generated by uploading all the 102 overlapped targets to the STRING database to visualize the interactions among them. After excluding the unconnected nodes, the PPI network consisted of 101 nodes connected by 872 edges (Figure 2C). This network was imported to Cytoscape software 3.10.4 for further analysis. After median filtration, the CytoNCA app was used to rank the 53 targets based on Betweenness and Closeness after they were filtered by the degree value (Supplementary Table S6). After that, the AMB-targets network of the top 20 hub targets (Table 1; Figure 2D) was built using Cytoscape (Figures 2E,F). PPARG was not the highest-degree node in the PPI network. Rather, it was selected for experimental assessment as a biologically plausible network-associated target based on its inclusion in the overlapping target set and its connectivity within the PPI network.

**TABLE 1:** The top 20 hub targets ranked by Degree method.

Rank	Target name	Degree
1	IL6	48
2	TNF	47
3	IL1B	45
4	TP53	44
5	MMP9	43
6	CASP3	42
7	BCL2	41
8	NFKB1	41
9	EGFR	40
10	PTGS2	40
11	MMP2	36
12	PPARG	36
13	ICAM1	34
14	VCAM1	29
15	CASP1	28
16	RELA	27
17	PPARA	26
18	MAPK8	25
19	MDM2	24
20	MMP3	24

#### 3.1.5 Enrichment analysis of the related proteins

The 20 hub genes targets were submitted to the ShinyGO analysis tool for GO and KEGG enrichment analyses to confirm the significant functional and biological characteristics of the 20 disease-drug- related proteins. The analyses were performed with a P value <0.05 in BP, CC, and MF (Supplementary Table S7). The hub targets were enriched in the response to lipid, response to abiotic stimulus and response to oxygen-containing compound on the BP level. Additionally, top CC included Nf-Kappa b complex, membrane raft, membrane microdomain and Transcription regulator complex, while top MF involved phosphatase binding, protein domain specific binding and enzyme binding (Figures 3A–C).

**FIGURE 3:**
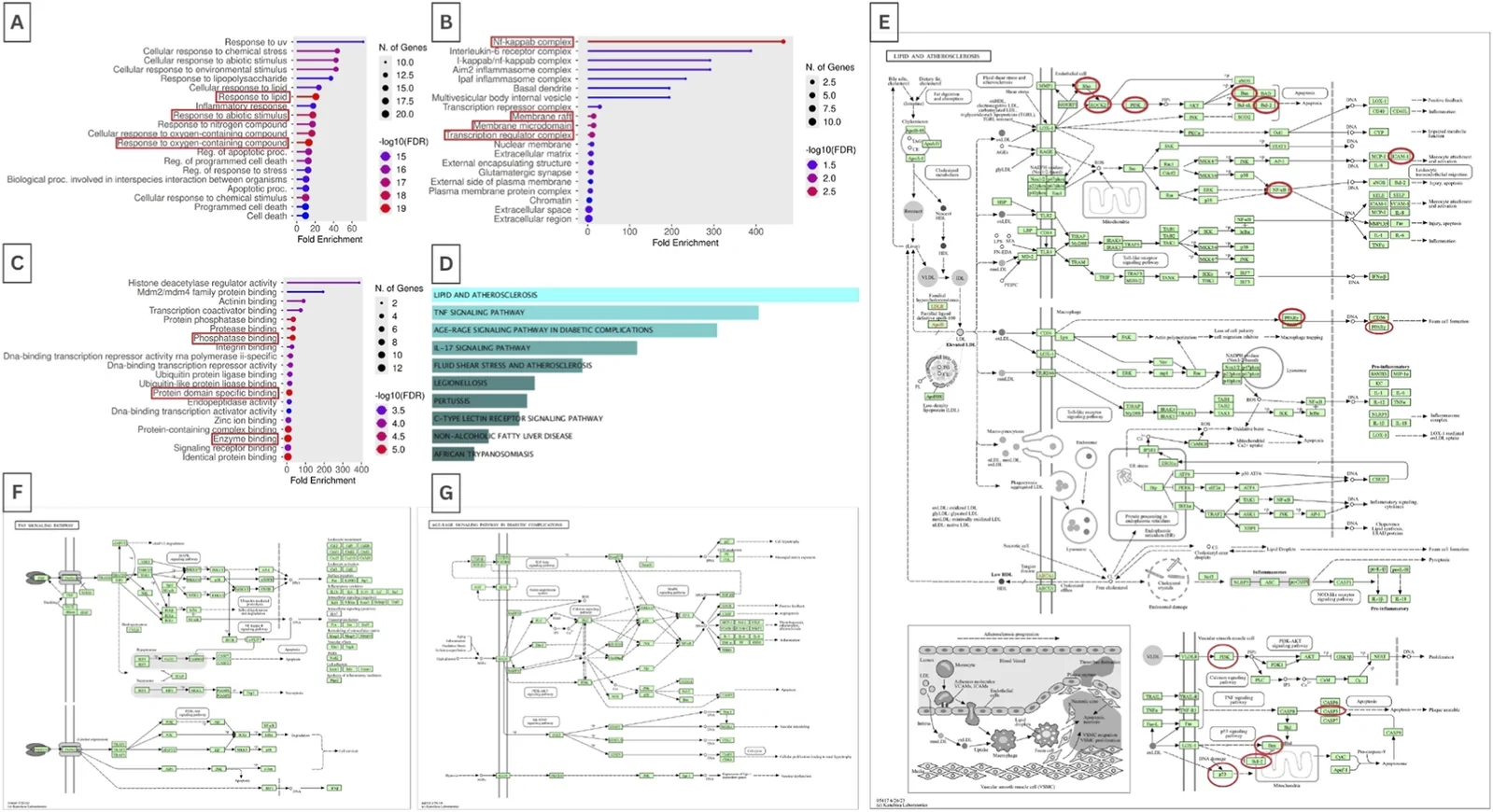
ShinyGO enrichment analysis. **(A-C)** Top 20 significantly enriched Gene Ontology (GO) analyses (sorted by Fold Enrichment) including biological process (BP), cellular component (CC), and molecular function (MF), respectively, **(D)** Top 10 significantly enriched KEGG pathways (sorted by Fold Enrichment), **(E)** The lipid and atherosclerosis pathway, nodes corresponding to genes from our overlapping target set and functionally related components are circled red. **(F)** TNF signaling pathway and **(G)** AGE-RAGE signaling pathway in diabetic complication.

To identify the likely pathways associated with the impact of AMB against MTX-induced kidney injury, a KEGG pathway analysis was performed (P value level set <0.05). The lipid and atherosclerosis, TNF signaling pathway and AGE-RAGE signaling pathway in diabetic complication were identified among the top 10 KEGG Pathways involved in the pathophysiology of AKI (Supplementary Table S7; Figure 3D). Significantly, these enriched pathways included a strong linkage between RhoA/ROCK, PI3K, ICAM-1, PPARG, NF-κB, Bax and Bcl-2 (Figures 3E–G). These targets were further selected to be validated experimentally in association with other markers involved in AKI pathogenesis.

### 3.2 Effect of ambrisentan on histological aspects

As illustrated in Figure 4, H&E-stained sections of the renal cortex from the normal control group exhibited normal renal architecture. The renal corpuscles, along with the proximal and distal convoluted tubules (PCTs and DCTs), appeared normal. The PCTs were lined by pyramidal cells, while the DCTs were surrounded by cuboidal cells. Similarly, sections from the AMB group displayed an unaltered renal cortical structure, comparable to the control group. In contrast, the MTX group demonstrated pronounced histological changes confirmed by significant difference in histological score from the control sections. Several renal corpuscles appeared distorted with widened capsular spaces, while others presented congested intraglomerular capillaries with narrowed capsular spaces. There was notable cytoplasmic vacuolation in the epithelial lining of PCTs and DCTs, along with evidence of blood vessel congestion and marked lymphocytic infiltration. However, the MTX + AMB group exhibited substantial improvement in histological score compared to MTX group, with the renal cortex largely returning to its normal architecture. The glomeruli and renal tubules resembled those of the control group, indicating a near-complete recovery. These results were furtherly confirmed in all groups by scoring the severity of histopathological alterations in the kidneys (Table 2).

**FIGURE 4:**
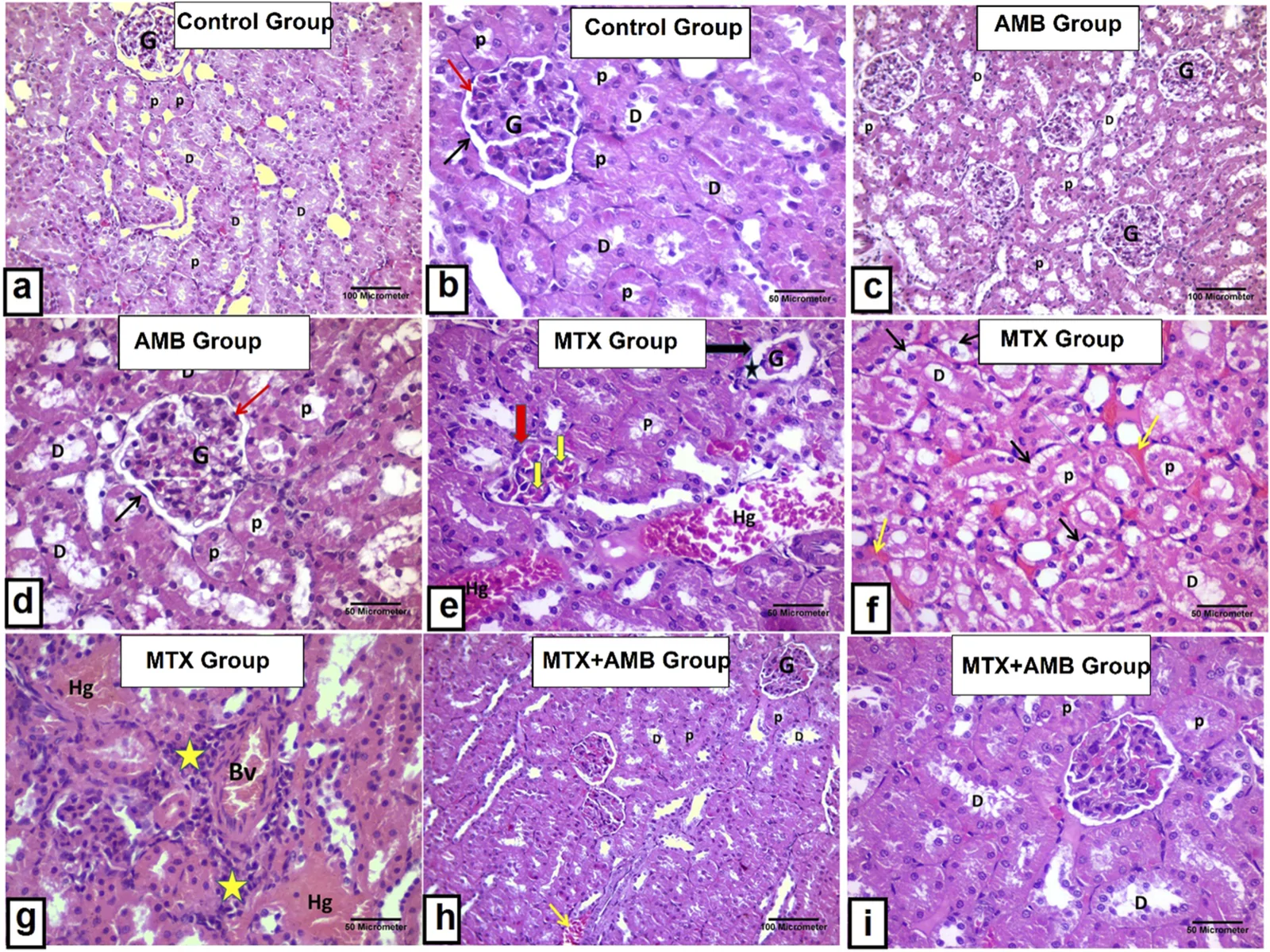
Representative photomicrographs of H&E-stained renal cortex sections from experimental groups. [**(a,c,h)**: x200 and scale bar = 100 μm, **(b,d,e,f,g,i)**: x400 scale and bar = 50 μm]. Control group **(a,b)**: showing normal renal architecture consists of a glomerulus (G), containing a tuft of capillaries surrounded by parietal simple squamous epithelial (black arrow) and visceral (red arrow) layers of Bowman’s capsule with normal Bowman’s space. PCTs (P) are lined by pyramidal cells with central rounded vesicular nuclei. DCTs (D) have a wider lumen, more cubical cells and rounded nuclei. AMB group **(c,d):** showing the same histological structure of renal cortex as control group. MTX group **(e,f,g)**: showing loss of normal renal architecture. **(e)** Distorted glomeruli (black arrow) with dilated Bowman’s space (black star), other glomeruli (red arrow) appear with intraglomerular congestion (yellow arrows) and multiple areas of hemorrhage (Hg) between renal tubules. **(f)** massive vacuolation of the cytoplasm of both proximal (P) and distal (D) convoluted tubules (black arrows) with congested intertubular capillaries (yellow arrows). **(g)** Large congested dilated blood vessel (Bv) and massive lymphocytic infiltration (yellow star) around congested dilated blood vessel (Bv) and between renal tubules. MTX + AMB group **(h,i)**: showing, normal renal histoarchitecture formed of glomeruli (G), proximal (p) and distal (D) convoluted tubules with few congested capillaries between renal tubules (yellow arrow).

**TABLE 2:** The effect of AMB on kidney function and histopathological changes in MTX induced AKI.

Groups	H&E (score of histopathological changes)	PAS stain (optical density)	PAS stain (tubular injury score)	Urea (µmol/mL)	Creatinine (nmol/mL)
Control	0.5 (1)	0.19 ± 0.02	0 (0)	4.8 ± 0.2	0.5 ± 0.02
AMB	1.0 (1)	0.18 ± 0.02^b^	0 (0)	5.4 ± 0.8	0.59 ± 0.03
MTX	3.5 (1)^a^	0.07 ± 0.02^a^	4 (1.25)^a^	15.5 ± 1.1^a^	1.63 ± 0.15^a^
MTX + AMB	1.0 (0.75)^a,b^	0.18 ± 0.01^a,b^	1 (0)^b^	7.6 ± 1.0^a,b^	0.9 ± 0.07^a,b^

For the histological score and PAS, stain tubular injury score: Data are expressed as median (interquartile range) (n = 6). a or b: statistical difference versus control or MTX, group., respectively at P < 0.01, using Kruskal–Wallis test followed by Dunn’s multiple comparison test. For PAS, stain optical density, Urea and creatinine: Data are expressed as mean ± SD (n = 6). a or b: statistical difference versus control or MTX, group., respectively at P < 0.001, using One way ANOVA, followed by Tukey’s multiple comparison test. AMB: ambrisentan; MTX: methotrexate.

As indicated in Figure 5, PAS staining of the control sections revealed a positive reaction in the renal corpuscles, a thin and uniformly regular Bowman’s capsule, thin regular basement membrane of tubules, and the apical brush border of PCTs (Figure 5a). The AMB group exhibited a PAS reaction that was largely comparable to the control group (Figure 5b). In contrast, sections from the MTX group displayed thickened basement membranes in the Bowman’s capsule and some renal tubules, along with disruption or even a complete absence of the apical brush border in the PCTs (Figure 5c). MTX caused a marked decline in the optical density by 0.6-fold compared to control sections with notable deterioration in tubular injury score (Table 2). However, sections from the MTX + AMB group showed results nearly comparable to those of the control group, with PAS-positive reactions observed in the glomerulus and tubules in a manner similar to the control findings (Figure 5d). Additionally, Table 2 showed a significant increase in optical density by 1.6-folds upon concurrent administration of AMB with MTX with marked improvement in tubular injury score.

**FIGURE 5:**
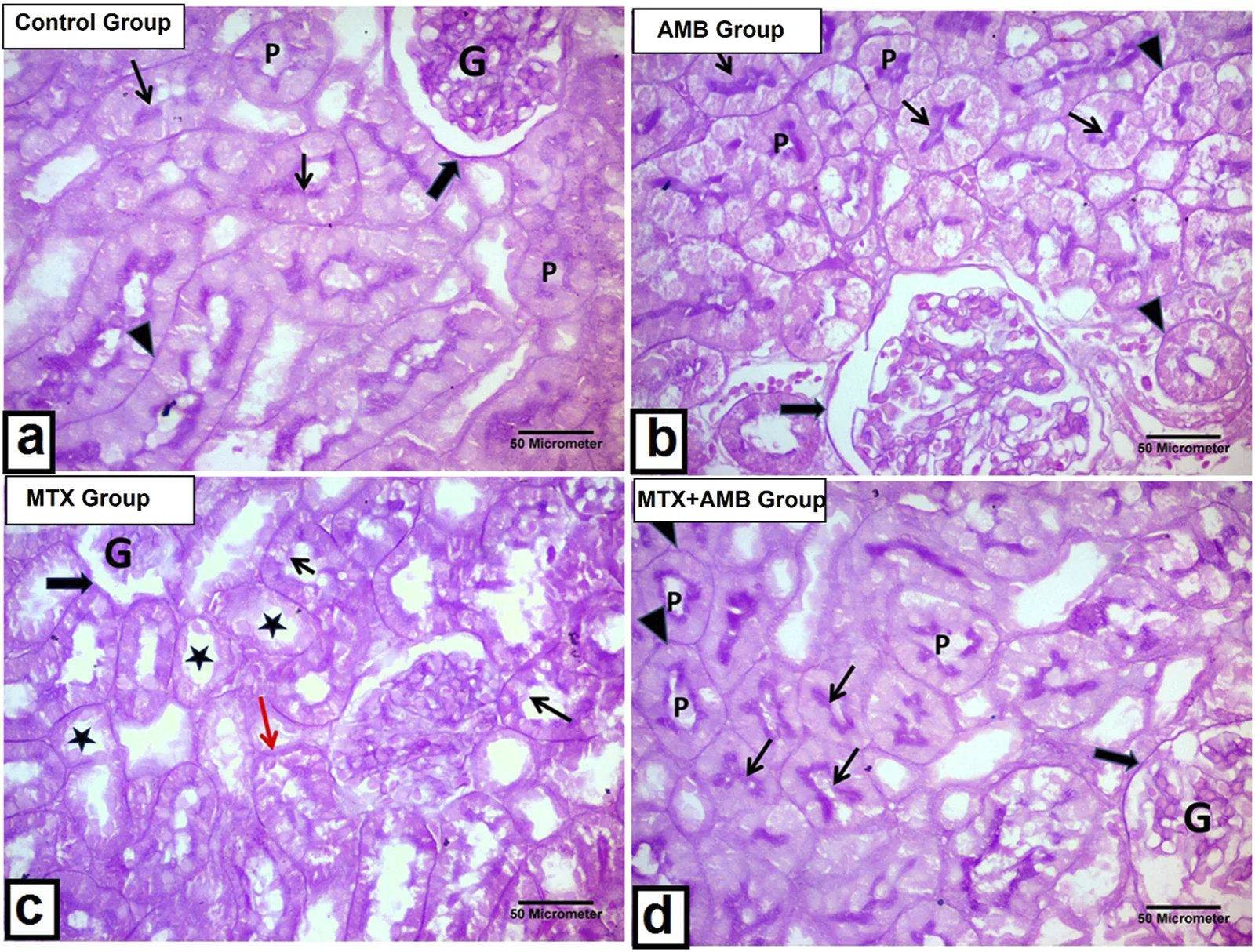
Photomicrographs of PAS-stained renal cortex sections from different experimental groups (x400, Scale bar = 50 μm). Control group **(a)** showing strong PAS-positive reaction in the glomeruli (G), at regular capsular (thick arrow), basement membranes of renal tubules (arrowhead) and the intact apical brush borders (thin arrow) of the PCTs (P). AMB group **(b)** showing PAS-positive reaction the same as that of control group. MTX group **(c)** showing thick basement membranes of the parietal layer of Bowman′s capsule (thick arrow) and renal tubules (red arrow). Interrupted apical brush borders of PCTs (P) (thin arrow) or even lost (stars). MTX + AMB group **(d)** showing restoration of the thin and regular capsular (thick arrow), glomerular (G) and tubular (arrowhead) strong PAS-positive reaction and the intact apical brush borders (thin arrow) of the PCTs (P).

### 3.3 Effect of ambrisentan on kidney function markers

In Table 2, rats treated with AMB alone showed normal kidney function. Meanwhile, MTX caused a marked deterioration in kidney function, with urea and creatinine rising by approximately 2.2 and 2.3-folds, respectively, relative to the control group. AMB cotreatment substantially mitigated this effect, lowering both urea and creatinine by about 0.5 and 0.4-folds, respectively, compared with the MTX group.

### 3.4 Effect of ambrisentan on kidney injury markers

In order to assess the severity of renal injury, KIM-1 and NGAL markers were employed. Herein, MTX administration resulted in marked elevations in renal KIM-1 and NGAL by almost four and 5-folds., respectively when compared to control rats. Adversely, concurrent treatment with AMB led to notable decline by 70% and 45% in both KIM-1 and NGAL., respectively markers in relation to MTX treatment (Table 3).

**TABLE 3:** The effect of AMB on kidney injury and oxidative stress markers in MTX induced AKI.

Groups	KIM-1 (ng/mg. Protein)	NGAL (ng/mg. Protein)	SOD (U/mg protein)	MDA (nmol/mg protein)
Control	0.3 ± 0.1	1.2 ± 0.2	2.12 ± 0.15	0.23 ± 0.05
AMB	0.4 ± 0.1	1.3 ± 0.2	2.05 ± 0.11	0.3 ± 0.06
MTX	1.7 ± 0.1^a^	2.9 ± 0.3^a^	0.67 ± 0.13^a^	1.34 ± 0.05^a^
MTX + AMB	0.5 ± 0.0^a,b^	1.6 ± 0.2^a,b^	1.93 ± 0.05^b^	0.56 ± 0.03^a,b^

Data are expressed as mean ± SD (n = 6). a or b: statistical difference versus control or MTX group., respectively at P < 0.001, using One way ANOVA, followed by Tukey’s multiple comparison analysis. AMB: ambrisentan; MTX: methotrexate.

### 3.5 Effect of ambrisentan on redox balance

MTX provoked oxidative damage indicated by lipid peroxidation and antioxidant status associated with AKI. MTX significantly increased MDA levels by 4.7-fold and decreased SOD activity by 0.7-fold versus control. AMB cotreatment produced a marked antioxidant effect, evidenced by reduced MDA by 0.6-fold, while SOD activity increased by 1.9-fold, compared with the MTX group (Table 3).

### 3.6 Effect of ambrisentan on ICAM-1, PPAR-γ, NF-κB markers

Figure 6 shows that MTX markedly elevated renal ICAM-1 and NF-κB protein levels, by approximately 3.2 and 3.1-folds, respectively, versus controls. On the contrary, PPAR-γ was notably declined in MTX treated rats by 0.7-fold. Cotreatment with AMB substantially attenuated these MTX-induced changes, through lowering ICAM-1 and NF-κB by about 0.6 and 0.7-fold, respectively, while restoring normal levels of PPAR-γ.

**FIGURE 6:**
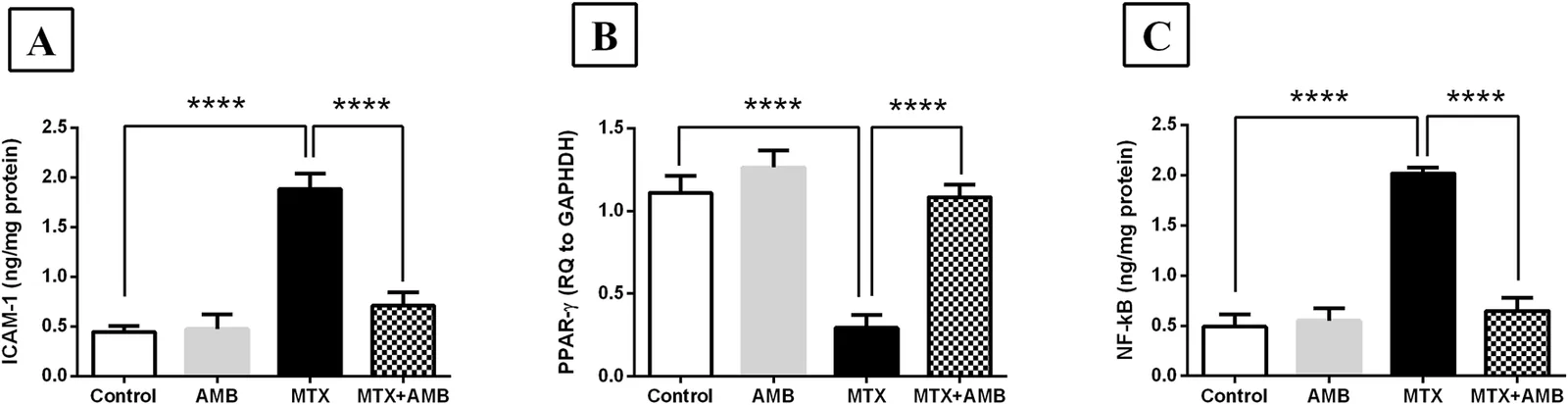
Effect of AMB on ICAM-1, PPAR-γ and NF-κB markers in MTX-induced AKI in rats. **(A)** ICAM-1, **(B)** PPAR-γ, **(C)** NF-κB. Data are presented as mean ± SD (n = 6). *p < 0.05, **p < 0.01, ***p < 0.001, and ****p < 0.0001 indicate statistical significance using One-way ANOVA followed by Tukey’s *post hoc* test. AMB: Ambrisentan; MTX: methotrexate.

### 3.7 Effect of ambrisentan on RhoA/ROCK signal

MTX administration caused a pronounced elevation in RhoA and its downstream target ROCK by approximately 1.9 and 3.2-folds, respectively, compared with control rats. Cotreatment with AMB significantly restored both markers, declining RhoA and ROCK expression by about 50% and 60%, respectively, relative to the MTX group (Figure 7).

**FIGURE 7:**
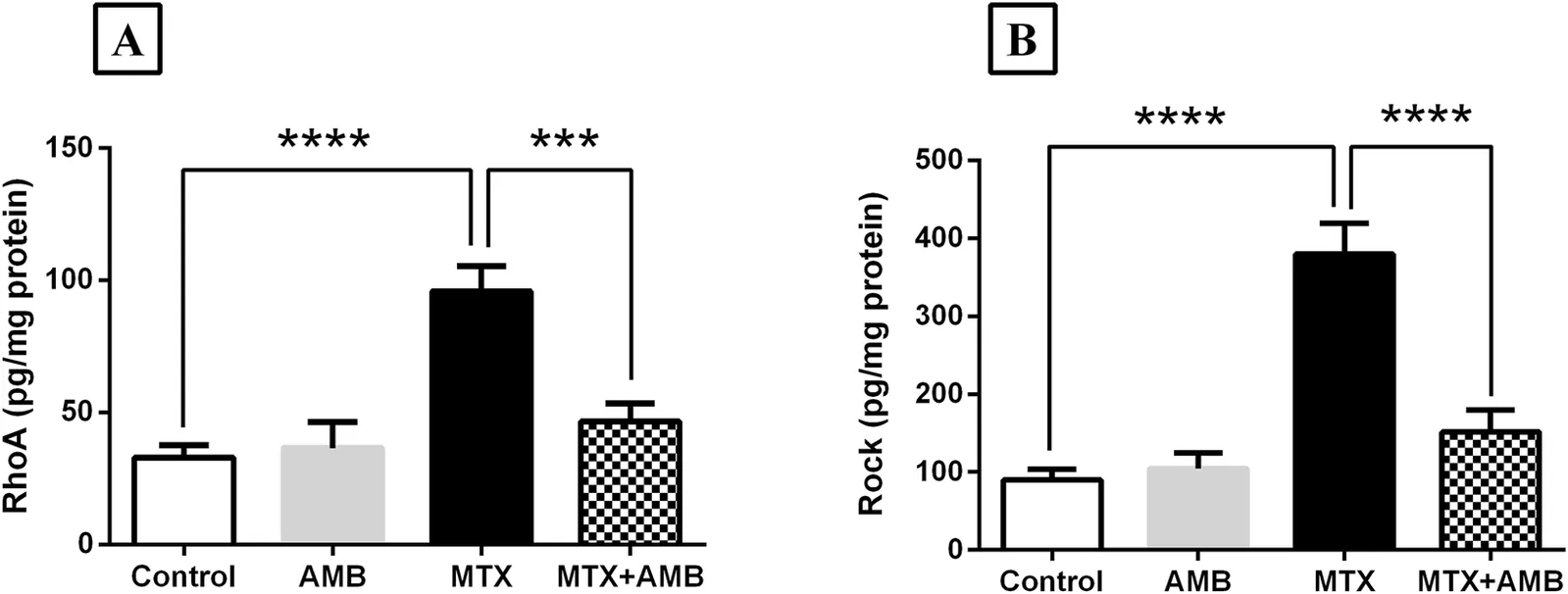
Effect of AMB on RhoA/ROCK signal in MTX-induced AKI in rats. **(A)** RhoA, **(B)** ROCK. Data are presented as mean ± SD (n = 6). *p < 0.05, **p < 0.01, ***p < 0.001, and ****p < 0.0001 indicate statistical significance using One-way ANOVA followed by Tukey’s *post hoc* test. AMB: Ambrisentan; MTX: methotrexate.

### 3.8 Effect of ambrisentan on PI3K, MDM2

Figure 8 displays a significant decrease in renal PI3K protein content in MTX treated rats by 36%, relative to control rats. AMB co-administration resulted in a notable restoration of PI3K by 45% compared with the MTX group. Similarly, MDM2 renal content demonstrated a significant fall in MTX-treated group to 0.4-fold of the non-treated group. Meanwhile, co-treatment with AMB markedly reelevated renal MDM2 to reach 1.61-folds of the MTX-administration.

**FIGURE 8:**
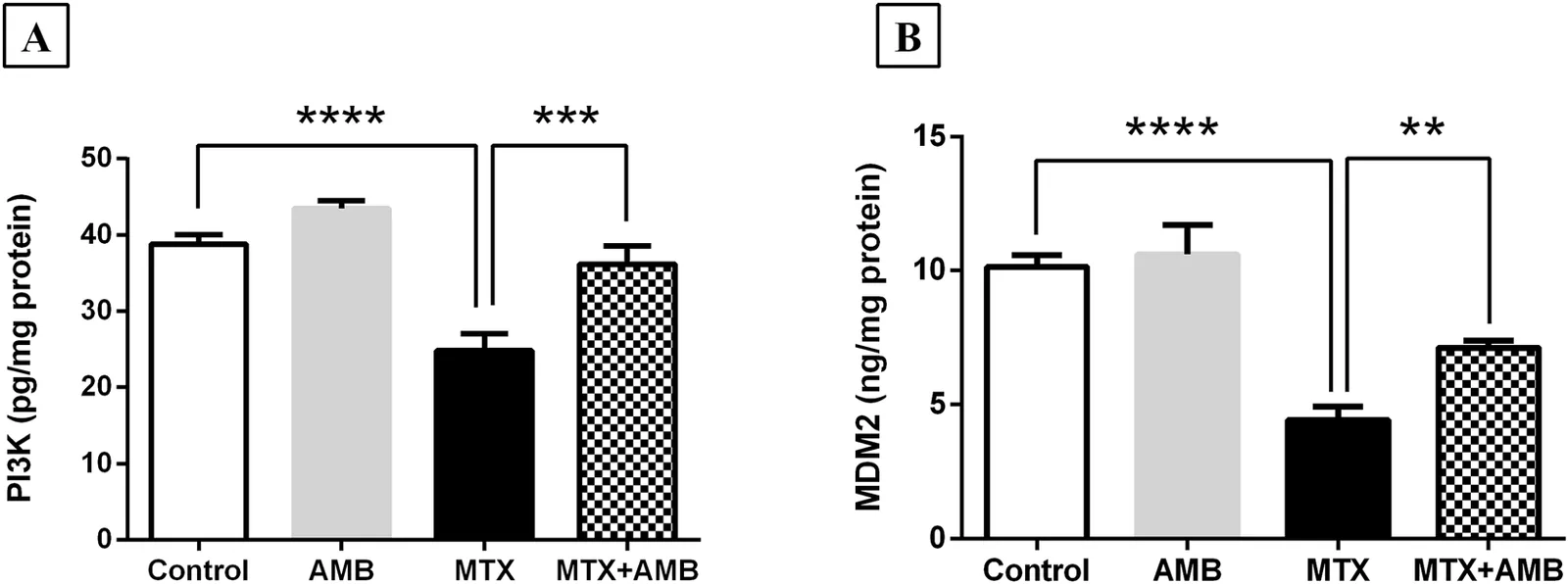
Effect of AMB on PI3K and MDM2 in MTX-induced AKI in rats. **(A)** PI3K, **(B)** MDM2. Data are presented as mean ± SD (n = 6). *p < 0.05, **p < 0.01, ***p < 0.001, and ****p < 0.0001 indicate statistical significance using One-way ANOVA followed by Tukey’s *post hoc* test. AMB: Ambrisentan; MTX: methotrexate.

### 3.9 Effect of ambrisentan on apoptotic markers

As indicated in Figures 9A,B,E, Bax and Bcl-2 produced distinct brown cytoplasmic reactions during immunohistochemical analysis. MTX significantly elevated the mean area of Bax-immunopositivity in both renal glomerular and tubular cells by 9-fold when compared to the control and AMB-treated groups. Notably, this increase was substantially counteracted by AMB treatment, as observed in the MTX + AMB group, where renal tissue exhibited a visible reduction in mean area of Bax-immunopositivity by 0.9-fold. Conversely, the MTX group showed a marked decline in the mean area of Bcl-2- immunopositivity within the glomerular and tubular regions by 0.8-fold compared to the control group. Importantly, AMB treatment effectively mitigated the MTX-induced decrease in Bcl-2 expression in renal tissue by almost 3.6-fold.

**FIGURE 9:**
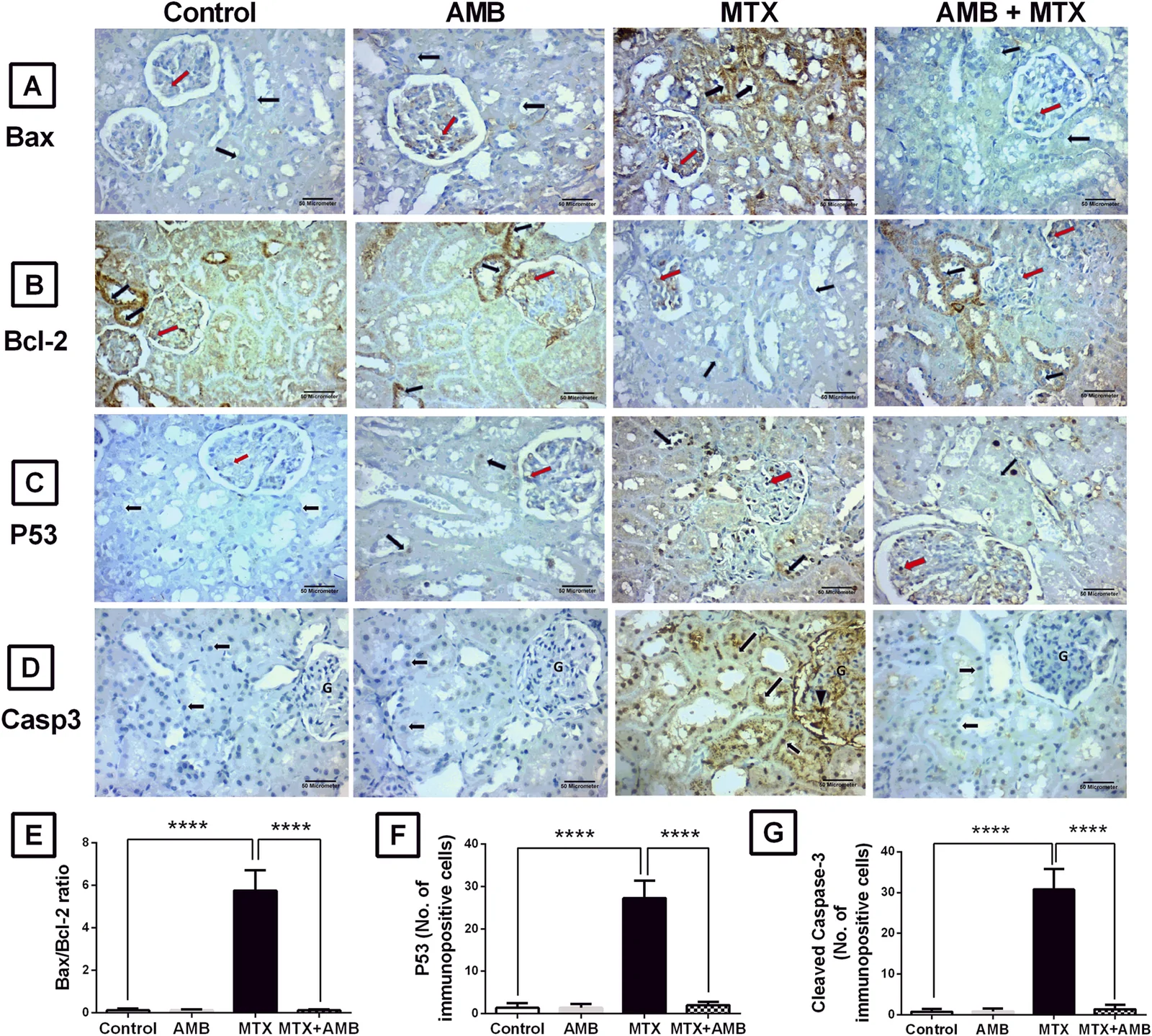
photomicrographs of immunohistochemical staining of Bax, Bcl-2, P53 and cleaved Caspase-3 of renal cortex sections of different experimental groups (x400). Photomicrographs (x400) of immunohistochemical staining of **(A)** Bax, **(B)** Bcl-2 **(C)** P53 and **(D)** cleaved Caspase-3. Control and AMB groups: showing scanty Bax immunopositive cells, abundant Bcl-2 immunoreactions, few P53 immunopositive cells in glomerulus (red arrow) and renal tubules (black arrow) and negative immunoreaction for cleaved caspase-3 in glomerulus **(G)** and renal tubules (black arrows). MTX group: showing extensive Bax immunopositivity, an apparent decline in Bcl-2 immunoreactions, abundant P53 and Caspase-3 immunopositive cells in glomeruli (red arrow and arrow heads) and renal tubular cells (black arrow). MTX + AMB group: showing weak Bax immunopositivity, extensive Bcl-2 immunoreactivity, few P53 immunopositivity in glomerular (red arrow) and tubular cells (black arrow) more or less as control groups and negative immunoreaction for cleaved Caspase-3 in the glomerulus **(G)** and most of renal tubular cells (black arrows) similar to the control group. **(E)** Graphical representation of Bax/Bcl-2 ratio. **(F)** Graphical representation of P53 immunopositive cells number **(G)** Graphical representation of Cleaved Caspase-3 immunopositive cells number. Data are presented as mean ± SD (n = 6). *p < 0.05, **p < 0.01, ***p < 0.001, and ****p < 0.0001 indicate statistical significance using One-way ANOVA followed by Tukey’s *post hoc* test. AMB: Ambrisentan; MTX: methotrexate.

The expression of p53 was assessed using immunohistochemical staining. In the control and AMB groups, p53 expression in the nuclei and cytoplasm of glomerular and tubular cells exhibited a faint immune reaction. Conversely, the MTX group demonstrated strong positive immunoreactions for p53, both in the tubular cells and the glomerulus that reached almost 20-folds of control group. On the other hand, the MTX + AMB group displayed minimal immunoreaction for p53 in the majority of glomerular and renal tubular cells, although a few tubular cells showed positive immunoreactions, which attained about 7% of MTX immunoreaction (Figures 9C,F).

Cleaved caspase-3 immunohistochemistry further confirmed the involvement of apoptotic execution in MTX-induced renal injury. Control and AMB-treated groups showed negative immunoreactivity for cleaved caspase-3 in the renal tissue. In contrast, the MTX group exhibited strong positive immunoreactivity in renal tubular cells and glomeruli, reaching almost 41-folds of control group. Notably, AMB co-administration markedly attenuated cleaved caspase-3 immunoreactivity, with an approximately 96% reduction in cleaved caspase-3-positive cells compared with the MTX group (Figures 9D,G). These findings provide additional evidence of enhanced apoptotic activity following MTX administration and its attenuation by AMB.

## 4 Discussion

MTX is an established cytotoxic antifolate agent widely used in oncology and inflammatory diseases, yet its therapeutic utility is constrained by its nephrotoxic potential. Renal injury associated with MTX may vary from mild subclinical tubulopathy to overt AKI. These limitations underscore the ongoing need to identify effective reno-protective adjunct therapies and preventive strategies to preserve renal function during MTX treatment. To the best of the authors’ knowledge, this is the first study to demonstrate the protective effects of AMB, a selective ETAR antagonist, against MTX-induced nephrotoxicity. Our results show that AMB pre-treatment significantly attenuates renal injury, highlighting its potential as a novel prophylactic reno-protective strategy in the context of MTX therapy. The findings suggest that the observed benefits are mediated through modulation of downstream signaling pathways, including inhibition of RhoA/ROCK and restoration of PPAR-γ and PI3K/MDM2-associated signaling, which collectively reduce oxidative stress, inflammation, and apoptosis.

Going through the current study, the histopathology showed that there was a significant damage to the renal cortex caused by MTX, with features such as distortion of glomeruli, modified Bowman’s space, vacuolation of tubular epithelium, vasocongestion, and inflammatory infiltrates. Our findings are consistent with earlier studies that implicate oxidative stress and inflammatory pathways in MTX-mediated renal damage (Troxell et al., 2016; Kutbi and Almalki, 2024). Tubular vacuolation probably represents changes resulting from oxidative damage, while vascular congestion and glomerular pathology might be due to ET-1-induced vasoconstriction and endothelial dysfunction (Kohan et al., 2011a). AMB pre-treatment markedly alleviated the histopathological alterations, restoring renal morphology to near-normal with a notable reduction in inflammatory infiltrates, an effect that is compatible with the known pharmacology of AMB, suggesting a link between ETAR blockade and the mitigation of vasoconstriction, inflammation, and oxidative stress (Ma et al., 2025).

Further evidence of the severity of the MTX-induced renal lesions was shown by the results of the PAS analysis. The rats that were administered MTX had thickened Bowman’s capsules and basement membranes of the tubules along with disruption and loss of the PAS-positive brush border of PCTs. The observed changes reflect damage to glomerular and tubular structures, corroborating earlier studies that implicate MTX in proximal tubular epithelial and basement membrane injury and in oxidative stress–mediated renal dysfunction (Troxell et al., 2016; Kutbi and Almalki, 2024). This brush border destruction is particularly important because it signifies proximal tubular injury. Given the predominant renal elimination of MTX, proximal tubules are particularly susceptible to drug and metabolite accumulation, which may contribute to MTX-induced tubular toxicity (Wiczer et al., 2016). The observed increase in basement membrane thickness may reflect acute structural alterations secondary to tubular injury, including epithelial swelling and disruption of tubular integrity, rather than established fibrotic remodeling, consistent with the pathological features of acute tubular injury following toxic renal insults (Wen et al., 2020). Most of the PAS response was normalized due to AMB pre-treatment, where Bowman’s capsule, tubular basement membranes, and proximal tubular brush border were intact. Therefore, AMB administration effectively prevented MTX-induced damage to both glomeruli and tubules. The mechanism underlying the reno-protective effect of AMB may include the reduction of oxidative stress and inflammation through inhibiting the expression of ET-1, as well as maintaining epithelial cell integrity (Komers and Plotkin, 2016; Ma et al., 2025).

Moreover, renal dysfunction was evident during MTX administration, as reflected by marked elevations in urea and creatinine levels, frequently measured renal physiological function indicators, besides elevated renal KIM-1 and NGAL levels, referring to the negative impact of MTX on renal filtration and proximal tubules (Gounden et al., 2026). Furthermore, the increase in KIM-1, a tubular injury marker, was supported by Kutbi and Almalki (Kutbi and Almalki, 2024), who reported the relation between elevated KIM-1 levels and the nephrotoxic impact of MTX on renal tubules. This observation is consistent with previous studies reporting that MTX nephrotoxicity arises from tubular injury, inflammation, and impaired filtration capacity (Howard et al., 2016; Wiczer et al., 2016). In addition, MTX disrupted the renal redox balance, demonstrated by reduced SOD activity and elevated MDA levels. Additional evidence that indicates the importance of the malfunctioning of antioxidant defenses for drug-induced nephrotoxicity is provided by cisplatin, which causes renal toxicity by inducing autophagy and the reduction in peroxiredoxin I (Park et al., 2024).

Similar alterations in oxidative stress markers have been repeatedly documented in experimental models of MTX-induced kidney injury (Vardi et al., 2008). AMB effectively corrected these alterations by lowering urea, creatinine, KIM-1 and NGAL levels, restoring SOD activity, and reducing MDA concentrations. The protective effect of AMB against kidney dysfunction may stem from its ability to counteract ET-1–mediated vasoconstriction, inflammation, and ROS generation (Kohan et al., 2011b; Saleh et al., 2011). This interpretation is further supported by experimental models of ischemia/reperfusion-induced AKI, in which inhibition of ET-1/ETAR signaling through ET-1 neutralization, ETAR antagonism, or suppression of endothelial ET-1 expression attenuated renal injury (Guan et al., 2015).

To clarify the mechanisms by which AMB alleviated nephrotoxicity and maintained renal functions, the present study utilized a network pharmacology strategy to forecast and examine the precise molecular targets and signaling pathways involved. Our network analysis identified 102 overlapping targets between AMB and MTX-induced kidney injury, demonstrating a significant intersection between the drug’s action and the disease’s pathophysiology. Further analysis of the PPI network identified top 20 hub targets, with PPAR-γ emerging as a network-associated target that was selected for experimental evaluation because it was present among the AMB–MTX–renal-injury overlapping hub targets, connected within the PPI network, and had a biologically plausible relationship to oxidative and inflammatory renal injury, which agree with the prespecified hypothesis that endothelin-related signaling may influence PPAR-γ activity. Simultaneously, KEGG enrichment identified lipid and atherosclerosis, TNF signaling, and AGE–RAGE signaling in diabetic complications as significantly enriched pathways. The lipid-and-atherosclerosis KEGG map includes multiple nodes functionally related to RhoA, ROCK and PI3K signaling. The network study established a strong correlation and linkage between these core targets and other key markers of inflammation and apoptosis, such as ICAM-1, PPAR-γ, NF-κB, Bax, and Bcl-2. These axes were therefore considered as biologically plausible downstream modules connected to the enriched pathways and were selected for experimental validation.

Moreover, the PPI interactions suggested that these targets function synergistically within a complex regulatory network to mitigate the oxidative-inflammatory and apoptotic cascades linked to MTX nephrotoxicity. Based on these preliminary findings from the network pharmacology analysis, we planned and conducted the *In-vivo* experiments to validate the hypothesis that AMB could preserve renal function and structure through the modulation of these specific signaling axes.

Several transcription factors linked to inflammatory mechanisms can be activated under conditions of oxidative stress. Specifically, MTX-induced ROS generation stimulates the ubiquitously expressed, redox-sensitive transcription factor NF-κB, which in turn upregulates TNF-α and IL-6, thereby amplifying the inflammatory response (Abd El-Twab et al., 2019). Furthermore, the regulation of ICAM-1 expression via the NF-κB signaling pathway has been extensively investigated across various contexts, and plays a central role in modulating inflammatory responses and immune function (Shen et al., 2021). This cascade amplifies leukocyte recruitment, vascular inflammation, and tissue injury, establishing NF-κB/ICAM-1 signaling as a central axis in MTX-induced nephrotoxicity. Consistent with the critical role played by inflammatory signaling suppression in drug-induced renal damage, ginsenoside Rg3 has been shown to alleviate cisplatin-induced nephrotoxicity via SIRT1-dependent inhibition of the NLRP3 inflammasome, thereby highlighting the importance of targeting inflammation pathways induced by oxidative stress for the treatment of nephroprotection (Zhai et al., 2026).

Conversely, PPAR-γ activation mitigates these effects by upregulating antioxidant enzymes and suppressing pro-inflammatory cytokine production. Evidence for the participation of PPAR-γ in the metabolic process of nephroprotection has been provided recently by the ability of Bavachin to mitigate the toxicity caused by cisplatin to the kidney through stimulation of mitochondrial β-oxidation and lipid metabolism via MFN2 (Luo et al., 2025). The importance of PPAR signaling as a renoprotective mechanism is further highlighted by studies showing that LTβR deletion confers protection against AKI by activating PPARα signaling, thus implying that PPARs' metabolic and inflammatory responses may form a conserved protective mechanism in renal injury (Wang Z. et al., 2024).

Its anti-inflammatory actions are partly mediated through interference with NF-κB signaling, thereby dampening downstream ICAM-1 expression and limiting inflammatory infiltration (Cunard et al., 2002). Importantly, the two mechanisms of endothelin signaling and PPAR-γ seem to have a functional relationship, since the enhanced signaling of ET-1 was linked with the inhibition of PPAR-γ activation (Wolf et al., 2014; Zhao et al., 2021). Therefore, the disruption of the ET-1/ETAR signaling pathway by AMB might alleviate the effect and activate the PPAR-γ responses. Here, the MTX-treated rats demonstrated a significant increase in renal NF-κB and ICAM-1 levels accompanied by a reduction in PPAR-γ expression, indicating a pronounced inflammatory response. This observation is consistent with previous studies reporting that MTX nephrotoxicity is mediated through NF-κB signaling pathways (Hassanein et al., 2023). In contrast, AMB administration significantly reduced ICAM-1 and NF-κB levels while upregulating PPAR-γ, thereby restoring the balance between pro- and anti-inflammatory signaling and maintaining basement membrane stability through the anti-inflammatory properties of PPAR-γ. These findings align with previous studies that have demonstrated the anti-inflammatory effects of AMB (Kawai et al., 2009; Kazimoglu et al., 2020; Almutary et al., 2025). However, it remains to be determined whether these effects also provide antifibrotic benefits in chronic kidney injury models.

Growing evidence indicates that ET-1 activates the RhoA/ROCK signaling cascade through ETA receptor engagement, establishing this pathway as a key downstream effector of ET-1 activity in renal pathology (Saleh et al., 2023). Our study showed a marked increase in RhoA and ROCK expression in renal tissue after MTX administration, a finding that aligns with the network analysis and previous reports implicating RhoA/ROCK signaling in renal inflammation, oxidative stress, and apoptosis (Komers, 2013). In addition, activation of RhoA/ROCK has been implicated in fibrotic signaling and extracellular matrix accumulation in chronic kidney disease models (Nagatoya et al., 2002), although its potential contribution to MTX-induced renal fibrosis warrants further investigation using collagen-specific staining in chronic nephrotoxicity models. Notably, pre-treatment with AMB significantly lowered RhoA and ROCK protein levels compared with MTX alone. This effect is likely due to ETAR blockade, since ET-1 is known to activate the RhoA/ROCK cascade, thereby linking AMB’s renoprotective effect to inhibition of this harmful pathway (Guan and Breyer, 2001; Kohan et al., 2011b; Komers, 2013).

Furthermore, excessive ROS generation and elevated pro-inflammatory cytokines frequently activate the mitochondrial apoptotic pathway (Zhang et al., 2025). Herein, immunohistochemical analysis revealed that MTX markedly promoted renal apoptosis, as evidenced by increased p53 and Bax expression, reduced Bcl-2 expression, and enhanced cleaved caspase-3 immunoreactivity. p53 is a major regulator of cell-cycle arrest, apoptosis, and cellular senescence. It participates in both the intrinsic and extrinsic apoptotic pathways (Devarajan, 2023), and is generally released in response to cellular stress. P53 activation has been associated with the pathogenesis of renal disorders, as demonstrated in multiple models of both AKI and chronic kidney disease (Overstreet et al., 2022). The observed increase in the Bax/Bcl-2 ratio is consistent with a shift toward mitochondrial apoptotic signaling, which can promote mitochondrial outer membrane permeabilization and cytochrome c release, followed by apoptosome formation, caspase-9 activation, and subsequent cleavage of procaspase-3 into active caspase-3. The increased cleaved caspase-3 immunoreactivity observed in the present study provides additional evidence of activation of the execution phase of apoptosis, complementing the observed alterations in the upstream apoptotic regulators Bax, Bcl-2, and p53. These findings are consistent with previous studies demonstrating that MTX-induced oxidative stress and inflammatory signaling converge on mitochondrial apoptotic pathways, thereby supporting the involvement of apoptosis in the pathogenesis of MTX-induced nephrotoxicity (Wei et al., 2021; Hassanein et al., 2023; Wasfey et al., 2023; Mishriki et al., 2024). This significance is further underlined by studies which have shown that TRPA1 protects the kidneys from damage due to contrast media by preserving mitochondrial dynamics via the AMPK/DRP1 pathway, thereby making mitochondrial quality control another significant factor in tubular cell survival in nephrotoxicity (Wang X. et al., 2024).

Meanwhile, AMB managed to reduce pro-apoptotic markers and increase Bcl-2. These results are in harmony with the previously reported anti-apoptotic effects of AMB against ischemia/reperfusion and other renal injury models through decreasing caspase-3 (Kazimoglu et al., 2020; Makled et al., 2025). This finding is consistent with previous reports highlighting the role of ET-1 in apoptosis-associated AKI (De Miguel et al., 2016). Accordingly, blocking ET-1 activity appears to be a rational approach for alleviating AKI by interrupting ET-1–mediated activation of the RhoA/ROCK and NF-κB pathways, thereby restoring PPAR-γ activity and promoting cellular survival (Hassanein et al., 2023).

In addition, the MDM2 and p53 axis forms a critical regulatory loop governing cell fate, specifically cell survival, senescence, and apoptosis in various forms of renal injury. Under basal conditions, MDM2 facilitates p53 proteasomal degradation, maintaining low levels of p53 to support tubular cell survival. However, under oxidative and metabolic renal stress, p53 accumulates, driving the transcription of pro-apoptotic gene Bax, and repressing the anti-apoptotic protein Bcl-2, which accelerates tubular cell apoptosis and exacerbates renal dysfunction (Brown et al., 2026).

Significantly, inhibition of RhoA/ROCK signaling is known to lead to the activation of the PI3K/Akt pathway (Medd et al., 2025), which suggests a possible mechanism by which the two signaling pathways discovered in the current study interact with each other. The activated Akt increases MDM2 phosphorylation, allowing for better p53 regulation and decreased apoptosis (Duan and Maki, 2016). Thus, the downregulation of RhoA/ROCK signaling following the administration of AMB may promote activation of PI3K/Akt/MDM2 signaling, resulting in decreased p53 apoptosis (Chibaya et al., 2021).

In line with earlier findings (Bagadood, 2025; Refaie et al., 2026), our study also revealed reduced expression of PI3K and MDM2 levels in renal cells post-MTX exposure, while AMB administration attenuated MTX-induced apoptosis and restored PI3K and MDM2 expression, indicating that changes in PI3K/MDM2-related signaling may contribute to its renoprotective effects. Although the present findings suggest the involvement of PI3K/MDM2-associated signaling in AMB-mediated nephroprotection, the absence of direct assessment of Akt and MDM2 phosphorylation represents a limitation of the current study. Further investigations incorporating pathway-specific activation markers are required to confirm the precise activation status and contribution of the PI3K/Akt/MDM2 signaling axis.

It is important to emphasize that AMB was administered prophylactically in the present study, beginning before the single MTX injection and continuing thereafter. This design establishes proof of concept for AMB as a preventive strategy rather than a treatment for established MTX-AKI. In clinical practice, high-dose MTX nephrotoxicity is strongly influenced by MTX/7-hydroxymethotrexate precipitation, renal clearance, hydration status, and pharmacokinetics (Faguer et al., 2025), and AMB would most plausibly be used either concurrently with MTX or as an early intervention upon detection of rising creatinine or tubular injury biomarkers. However, the present study did not determine whether AMB alters MTX exposure, renal elimination, or antineoplastic efficacy. Future studies should therefore evaluate AMB administered after MTX-induced injury has been established, as well as in chronic nephrotoxicity models, and should assess potential drug–drug interactions and preservation of MTX antitumor activity if a clinically relevant chemotherapy-adjunct claim is to be pursued.

In summary, the present study identifies AMB as a promising renoprotective agent capable of markedly mitigating MTX-induced kidney injury through a multifaceted mechanism involving selective ETA receptor blockade. Preserving renal architecture, improving functional biomarkers, and attenuating oxidative stress, inflammation, and apoptosis all indicate that AMB could interrupt core pathogenic pathways underlying MTX nephrotoxicity. Its beneficial actions appear to involve coordinated modulation of the PPAR-γ, RhoA/ROCK, and PI3K/MDM2 signaling axes, which provides a coherent mechanistic framework linking ET-1 antagonism to reduced vasoconstrictive, proinflammatory, and proapoptotic responses in the kidney. Nevertheless, considering the strong preclinical protection observed here and prior evidence of AMB renoprotective impacts, further studies are warranted to confirm these effects in chronic nephrotoxicity models and evaluate whether AMB can offer clinically meaningful renal protection during MTX therapy. Additionally, future studies are crucial to determine whether AMB alters MTX pharmacokinetics, renal elimination, or antineoplastic efficacy before any clinical adjunctive use can be considered.

## Data Availability

The data presented in the study are deposited in the Figshare repository, accession number: 10.6084/m9.figshare.34662099.

## Glossary

AKI: Acute kidney injury
AMB: Ambrisentan
ANOVA: Analysis of variance
Bcl-2: B-cell lymphoma 2
BP: Biological process
CMC: Carboxymethyl cellulose
CC: Cellular component
CTD: Comparative Toxicogenomics Database
DCTs: Distal convoluted tubules
ELISA: Enzyme-linked immunosorbent assay
EGFR: Epidermal growth factor receptor
ET-1: Endothelin-1
ETAR: Endothelin type A receptor
GAPDH: Glyceraldehyde-3-phosphate dehydrogenase
GO: Gene Ontology
H&E: Hematoxylin and eosin
HO-1: Heme oxygenase-1
I/R: Ischemia reperfusion
ICAM-1: Intercellular adhesion molecule-1
IL1B: Interleukin 1 beta
IL6: Interleukin 6
KIM-1: Kidney injury molecule-1
KEGG: Kyoto Encyclopedia of Genes and Genomes
MAPK8: Mitogen-activated protein kinase 8
MDM2: Mouse double minute 2
MDA: Malondialdehyde
MF: Molecular function
MMP: Matrix metalloproteinase
MTX: Methotrexate
NCBI: National Center for Biotechnology Information
NF-κB: Nuclear factor-kappa B
NGAL: Neutrophil gelatinase-associated lipocalin
NIGMS: National Institute of General Medical Sciences
OMIM: Online Mendelian Inheritance in Man
P53: Tumor protein p53
PAS: Periodic acid-Schiff
PCTs: Proximal convoluted tubules
PI3K: Phosphatidylinositol 3-kinase
PPAR-γ: Peroxisome proliferator-activated receptor gamma
PPI: Protein-protein interaction
ROCK: Rho-associated coiled-coil containing protein kinase
ROS: Reactive oxygen species
SOD: Superoxide dismutase
SEA: Similarity ensemble approach
STRING: Search Tool for the Retrieval of Interacting Genes/Proteins
TBS: Tris-buffered saline
TNF: Tumor necrosis factor
TP53: Tumor protein p53
VCAM1: Vascular cell adhesion molecule 1
