# Supplementary material for: Ambrisentan ameliorates methotrexate nephrotoxicity through modulation of RhoA/ROCK and PI3K/MDM2-associated signaling: network pharmacology-guided discovery and in vivo confirmation
Source: Front Pharmacol. 2026 Oct 29;17:1976309. doi: 10.3389/fphar.2026.1976309 (PMC13755328; doi:10.3389/fphar.2026.1976309)
Supplement: Supplementary file 1 [file Table1.docx]

**Supplementary Table S1: Chemical structure in SMILES format of Ambrisentan and Methotrexate from PubChem database:**

| Drug name | SMILES format |
| --- | --- |
| Ambrisentan | CC1=CC(=NC(=N1)O[C@H](C(=O)O)C(C2=CC=CC=C2)(C3=CC=CC=C3)OC)C |
| Methotrexate | CN(CC1=CN=C2C(=N1)C(=NC(=N2)N)N)C3=CC=C(C=C3)C(=O)N[C@@H](CCC(=O)O)C(=O)O |
